# Supplementary material for: Development and optimization of self-collected, field stable, saliva-based immunoassays for scalable epidemiological surveillance of pathogen-specific immunity
Source: medRxiv. 2026 Mar 6:2026.03.05.26347729. Preprint. [Version 1] doi: 10.64898/2026.03.05.26347729 (PMC13001388; doi:10.64898/2026.03.05.26347729)
Supplement: Supplement 1 [file media-1.pdf]

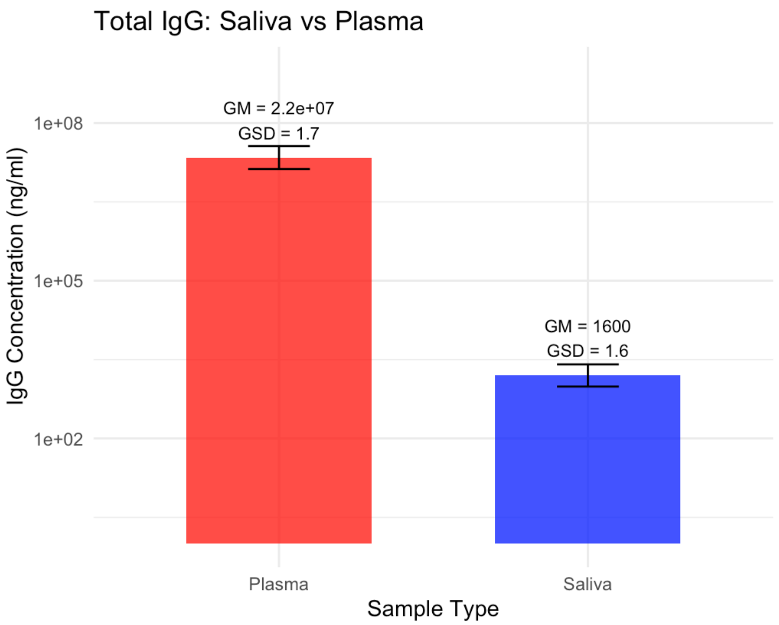

Supplemental Figure 1. Concentration of total IgG in serum and saliva as determined by ELISA

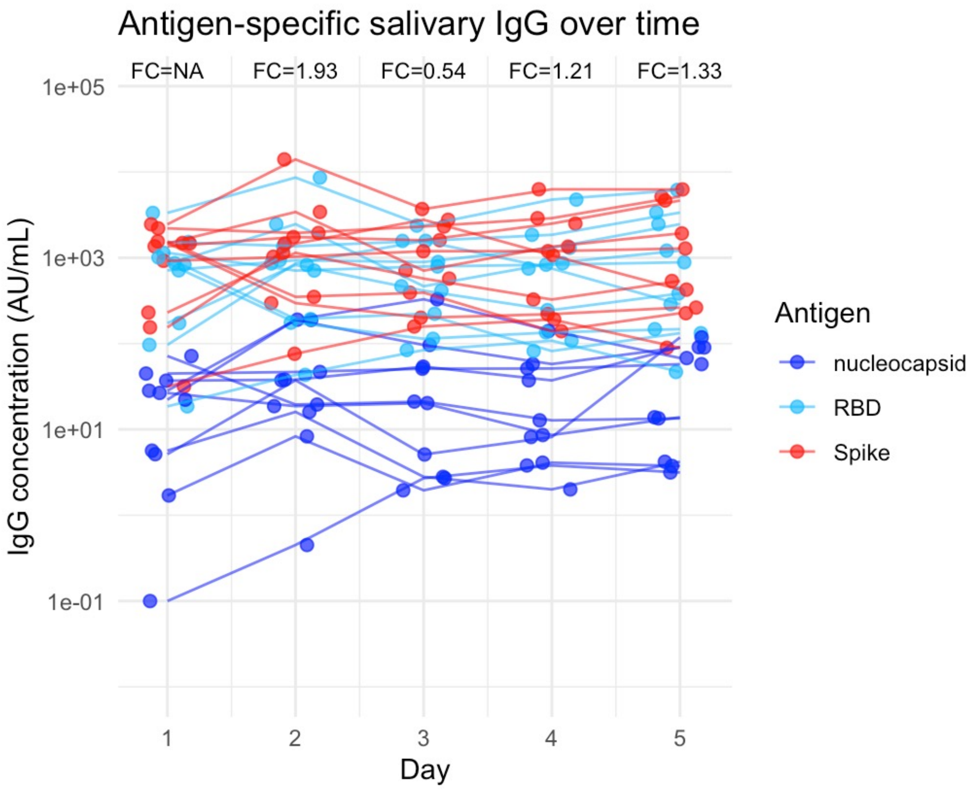

Supplemental Figure 2. Concentration of SARS-CoV-2 specific IgG in saliva collected over 5 consecutive days. Lines connect samples connected from the same individual on sequential days

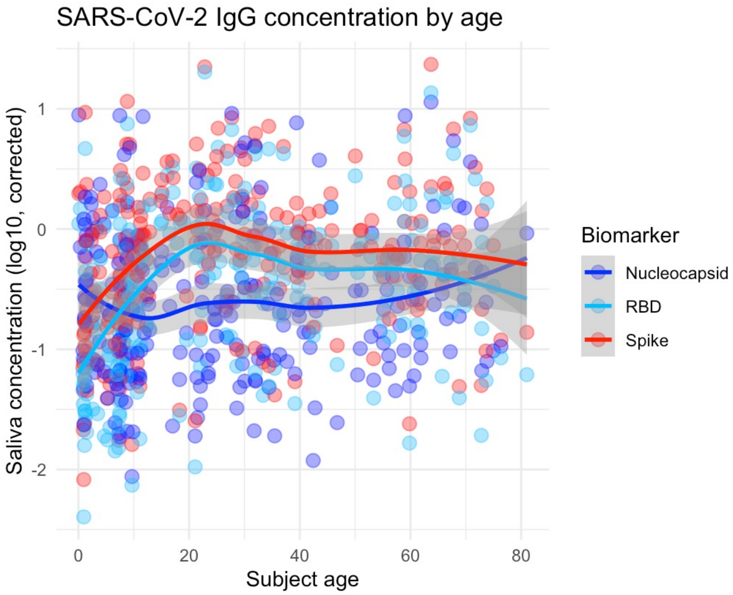

Supplemental Figure 3. Concentration of SARS-CoV-2 specific IgG in saliva corrected for total IgG concentration in saliva plotted against volunteer age

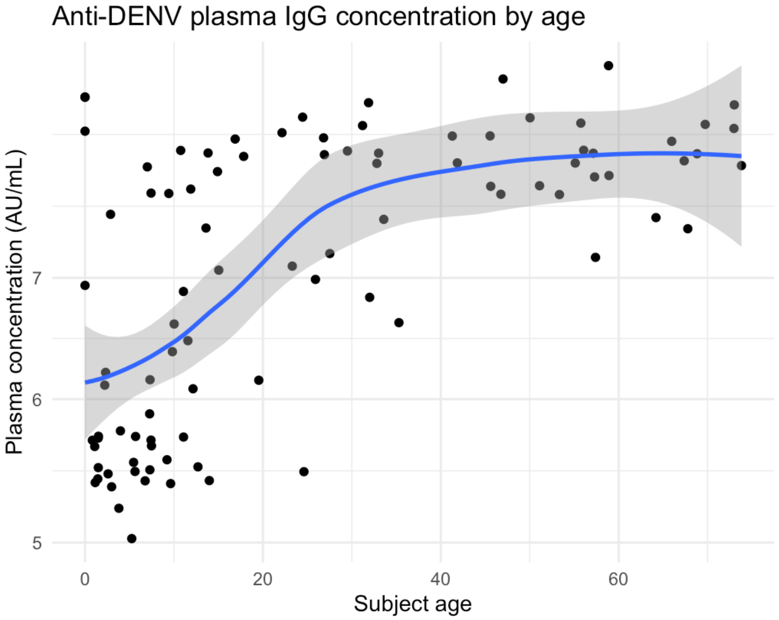

Supplemental Figure 4. Concentration of DENV NS1-specific IgG in plasma plotted against volunteer age

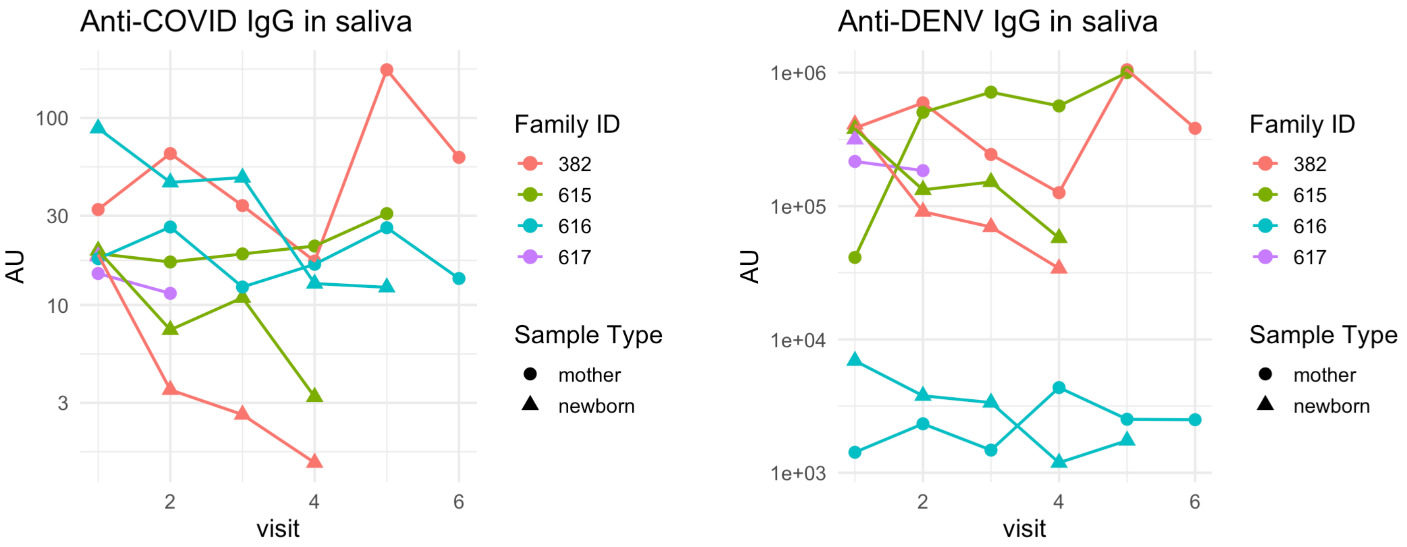

Supplemental Figure 5. Concentration of DENV NS1-specific IgG in collected from four mother/infant dyads plotted against the visit from which the same was collected
